# Low complexity symmetric-coded based sphere decoding for low-rate polar codes

**DOI:** 10.1038/s41598-023-28256-1

**Published:** 2023-01-21

**Authors:** Yuanbo Liu, Haiqiang Chen, Jichang Chen, Lanjuan Liao, Fuyi Huang, Youming Sun, Xiangcheng Li

**Affiliations:** 1 School of Computer, Electronics and Information, Guangxi University, Nanning, 530004 China; 2 Nanning University, Nanning, 530004 China; 3 Guangxi Key Laboratory of Multimedia Communications and Network Technology, Nanning, China

**Keywords:** Information theory and computation, Electrical and electronic engineering

## Abstract

The sphere decoding (SD) algorithm can provide (sub)optimal solutions with reduced computational complexity of maximum likelihood (ML) detection for multi-input multi-output (MIMO) communication systems. In this paper, we propose a novel low complexity symmetric-coded based SD algorithm for short polar codes with low rate. At the encoding stage, the first *N*/2 sub-channels transmit the frozen bits, while the information bits are selected from the latter *N*/2 sub-channels. Two symmetric codes are generated due to the mathematical structure of the generator matrix, which is well conditioned to the SD search. At the decoding stage, the presented SD algorithm computes the Euclidean distance value by the combined signals to estimate the latter *N*/2 input bits. Furthermore, the backtrack operation starts from the earlier $(N/2+1)$-th bit, which can significantly reduce the average visited nodes (AVN). Simulation results show that, compared to the original SD algorithm, the presented variant of the SD algorithm can reduce the AVN to $0.9\%$ for the polar code *P*(64, 14) at SNR = 1  dB with a performance loss within 0.2 dB. The presented SD algorithm may find applications in MIMO systems where the complexity of the standar ML detection increases exponentially with the transmitting antennas.

## Introduction

As the first class provably capacity-achieving channel codes, polar codes have been adopted as the coding standard for the uplink/downlink control channels for the enhanced mobile broadband (eMBB) scenario in 5th generation wireless communication systems^1,2^. Since the payload size of the control channel is relatively small, well-designed decoding algorithms for such short polar codes becomes necessary. Although the successive cancellation (SC) decoding has relative low complexity, it suffers from performance degradation, especially for short-to-medium code length. Two variants of the SC algorithm are developed to achieve the balance between the performance and the complexity, including the successive cancellation list (SCL) algorithm^3^ and the cyclic redundancy check aided SCL (CA-SCL) algorithm^4^. However, these two algorithms can not reach the maximum likelihood (ML) decoding performance for small/medium list size.

Different from the SC-based decoding algorithms, the sphere decoding (SD) algorithm can provide optimal or sub-optimal solutions with reduced computational complexity of the ML detection, which is commonly used for multi-input multi-output (MIMO) communication systems. Several SD-based decoding algorithms are designed for the MIMO systems, such as the single soft-input soft-output (SISO) SD algorithm^5^, the king sphere decoder (KSD)^6^ and its SISO detection algorithm^7^, which can make efficient trade-offs between complexity and performance. The polar-coded MIMO systems were investigated in ref.^8^, where the polar coding, modulation and MIMO-transmission are jointly optimized. The performance of polar-coded MIMO systems can be further improved by the unitary pre-coding scheme^9^.

The SD algorithm is proved to be an efficient decoding method for polar codes^10^. It can attain the optimal error performance with cubic complexity of $O(N^{3})$. The SD algorithm with fixed lower bounds and dynamic lower bounds is proposed in ref.^11^, which significantly reduces the complexity without performance loss. Furthermore, the list sphere decoding (LSD) is proposed in ref.^12^, taking the absolute value of received logarithmic likelihood ratio (LLR) as the substitution of Euclidean distance (ED). The LSD algorithm can match the error performance of the SC and SCL algorithms with lower complexity. Based on the work in ref.^12^, Zhou *et al.* proposed an improved software LSD with synchronous determination for the low-rate polar codes such as *P*(64, 7) or *P*(128, 8)^13^, which can achieve up to 2 dB performance gain. In ref.^14^, the authors combined CRC codes with SD algorithm and then proposed the CRC-Aided SD (CA-SD) by simplifying the check matrix ${\textbf {H}}_{CRC}$, which can reduce the complexity and has a comparable complexity with the CA-SCL.

In this paper, we propose a novel low complexity symmetric-coded based SD algorithm which chooses the information set only from the latter half of the sub-channels rather than the whole sub-channels. Two copies of symmetric-coded bits that have the same values can be generated according to the presented decoding strategy. In this way, the presented SD algorithm can stop decoding early when the ED of *N*/2 received signals are computed, which can greatly reduce the complexity. Numerical results show that the average visited nodes (AVN) of the presented SD algorithm can reduce to about $0.9\%$ at SNR = 1dB for the *P*(64, 14) polar code, with a performance loss within 0.2 dB. Furthermore, for the polar codes with lower rates, for example, the *P*(64, 8) polar code, the AVN reduces to about $2.6\%$ but the performance loss can be almost negligible. The proposed symmetric-coded based SD algorithm may find applications in MIMO systems where the complexity of the standard ML detection increases exponentially with the transmitting antennas.

The remainder of this paper is organized as follows. “Preliminaries” section derives the procedure of polar coding and describes the basic SD strategy. “The presented symmetric-coded based sphere decoding” section illustrates the proposed symmetric-coded based SD. “Performance and complexity evaluation” section analyses the performance and complexity. Finally, “Conclusion” section concludes the paper.

*Notation* Lower-case bold letters denote vectors with $c_i$ and $u_i$ denoting the *i*-th element of the vector ${\textbf {c}}_1^N$ and ${\textbf {u}}_1^N$, respectively; upper-case bold letters denote matrices with $g_{j,i}$ represents the element located in the *j*-th row and the *i*-th column of ${\textbf {G}}_N$; $D_i$ is the ED between $y_i$ and $x_i$ and $d_i^N$ represents the partial ED value between the vector $y_i^N$ and $x_i^N$; finally, ${\mathscr {A}}$ and ${\mathscr {A}}^c$ denote the information and frozen sets, respectively.

## Preliminaries

### Polar codes

The construction of polar codes depends on the *n*-th Kronecker product of the basic polarizing matrix $${\textbf {F}}=\left[ \begin{array}{cc} 1 &{} 0\\ 1 &{}1\\ \end{array}\right]$$, which can be represented as

1 $$\begin{aligned} {\textbf {G}}_{N}={\textbf {F}}^{\otimes n}, \end{aligned}$$

where ${\textbf {G}}_{N}$ is the *N*-dimension generator matrix with $N=2^{n}$. Let *P*(*N*, *K*) be a polar code with rate $R=K/N$, the encoding process can be defined as

2 $$\begin{aligned} {\textbf {c}}_1^N=u_1^N {\textbf {G}}_{N}={\textbf {u}}_1^N{\textbf {F}}^{\otimes n}, \end{aligned}$$

where ${\textbf {c}}_1^N=(c_1,c_2,\ldots ,c_N)$ is the encoded vector and ${\textbf {u}}_1^N=(u_1,u_2,\ldots ,u_N)$ denotes the input vector combined with *K* information bits and $N-K$ frozen bits. According to the polar encoding strategy, the *K* most reliable sub-channels with indices in ${\mathscr {A}}$ transmit information bits and the rest $N-K$ sub-channels with indices in ${\mathscr {A}}^c$ carry frozen bits, which are usually set to be zeros. The reliability of the sub-channel can be computed by the Bhattacharyya parameter^15^, density evolution^16^, Gaussian approximation (GA)^17^ or the polarization weight^18^for different types of channels. The design of the information set ${\mathscr {A}}$ and the frozen set ${\mathscr {A}}^c$ is of importance, since it will strongly impact the error performance of the resulting polar codes.

Without loss of generality, the binary input additive white Gaussian noise (BI-AWGN) channel and the binary phase-shift keying (BPSK) modulation are considered in this paper. Then the encoded vector $c_1^N=(c_1,c_2,\ldots ,c_N)$ is mapped to $x_1^N=(x_1,x_2,\ldots ,x_N)$ with $x_i=1-2c_i$, for $1\le i \le N$. After channel transmitting, the received vector is denoted by $y_1^N=(y_1,y_2,\ldots y_N)$ and each $y_i$ can be calculated by

3 $$\begin{aligned} y_i=x_i+n_i, \end{aligned}$$

where $1\le i \le N$ and $n_i$ represents the channel noise with mean 0 and variance $\sigma ^2$.

### Sphere decoding algorithm

The sphere decoding method for polar codes is equivalent to solve the minimization problem as follows.

4 $$\begin{aligned} {\hat{u}}_{1}^{N}=\mathop {argmin}\limits _{u_1^N\in \{0,1\}^N}||y_1^N-(1-2u_{1}^{N}{\textbf{G}}_N)||^{2}, \end{aligned}$$

where ${\hat{u}}_{1}^{N}$ is the estimated value. It is shown that, such decoding procedure is actually to enumerate all the possible input information vector. Let $D_i$ be the ED between $y_i$ and $x_i$, which can be calculated as

5 $$\begin{aligned} D_i=||y_i-x_i||^2=(y_i-(1-2\sum _{j=1}^N\oplus u_jg_{j,i}))^2, \end{aligned}$$

where $1 \le i \le N$ and $g_{j,i}$ represents the element located in the *j*-th row and the *i*-th column of ${\textbf {G}}_N$. Since ${\textbf {G}}_N$ is a lower triangular matrix, the value $D_i$ can be simplified as

6 $$\begin{aligned} D_i=(y_i-(1-2\sum _{j=i}^N\oplus u_jg_{j,i}))^2. \end{aligned}$$

Then the partial ED value between the vector $y_i^N$ and $x_i^N$ can be computed by

7 $$\begin{aligned} d_i^N=\sum _{k=i}^N D_k=\sum _{k=i}^N(y_k-(1-2\sum _{j=k}^N\oplus u_jg_{j,k}))^2, \end{aligned}$$

which can be recursively computed by satisfying the constraint

8 $$\begin{aligned} d_i^N = d_{i+1}^N + D_i \le r_0^2, \end{aligned}$$

where $1 \le i \le N-1$ and $r_0$ is the radius for the SD (initialized to maximum). It can be seen from Eq. (8) that, the SD can be regarded as the depth-first search algorithm in a backward manner, starting form the *N*-th bit to the first bit. After the first bit $\hat{u}_1$ is estimated, we get the whole ED value $d_1^N$. Then $r_0$ is updated to a smaller value and the SD search is re-performed with the updated radius. Note that, the backtrack operation (starting from the first bit) is required in the SD procedure if the value $d_i^N$ ( $2 \le i \le N$ ) is smaller than the current radius.

**Figure 1:**
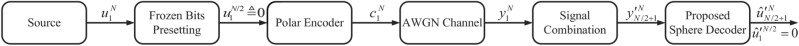
The system model.

## The presented symmetric-coded based sphere decoding

It is mentioned above that, the SD search requires the backtrack operation, which produces a great deal of computational complexity, especially for a large polar code length. To reduce the complexity, a symmetric-coded based SD algorithm is presented in this sub-section by the frozen bits presetting method. It is shown that, the resulting encoded bits are symmetric due to the mathematical structure of the generator matrix ${\textbf {G}}_{N}$. In this way, it is equivalently to shorten the block length, which can reduce the complexity caused by the backtrack operation. Meanwhile, two copies of the received signals can be exploited to mitigate the corruption caused by the channel noise when computing the ED value.

The key step to achieve the symmetric bits is to preset the frozen bits in polar encoding. Let ${\mathscr {A}}^c$ be the frozen set of the *P*(*N*, *K*) polar code with rate *R*. In the presented SD strategy, the former half of the sub-channels are all included in ${\mathscr {A}}^c$ . The system model of the presented algorithm is shown in Fig. 1. Let ${\mathscr {A}}^{c*}$ denote the set of the remaining frozen bit positions selected from the latter half of the sub-channels. Therefore, the frozen set ${\mathscr {A}}^c$ can be expressed as

9 $$\begin{aligned} {\mathscr {A}}^c = \{ 1,2, \ldots , N/2 \} \cup {\mathscr {A}}^{c*}, \end{aligned}$$

where ${\mathscr {A}}^{c*} \subseteq \{ N/2+1, N/2+2, \ldots , N \}$. Obviously, the code rate *R* is computed by

10 $$\begin{aligned} R = \frac{N/2 - |{\mathscr {A}}^{c*}|}{N} = \frac{1}{2} - \frac{|{\mathscr {A}}^{c*}|}{N}. \end{aligned}$$

**Figure 2:**
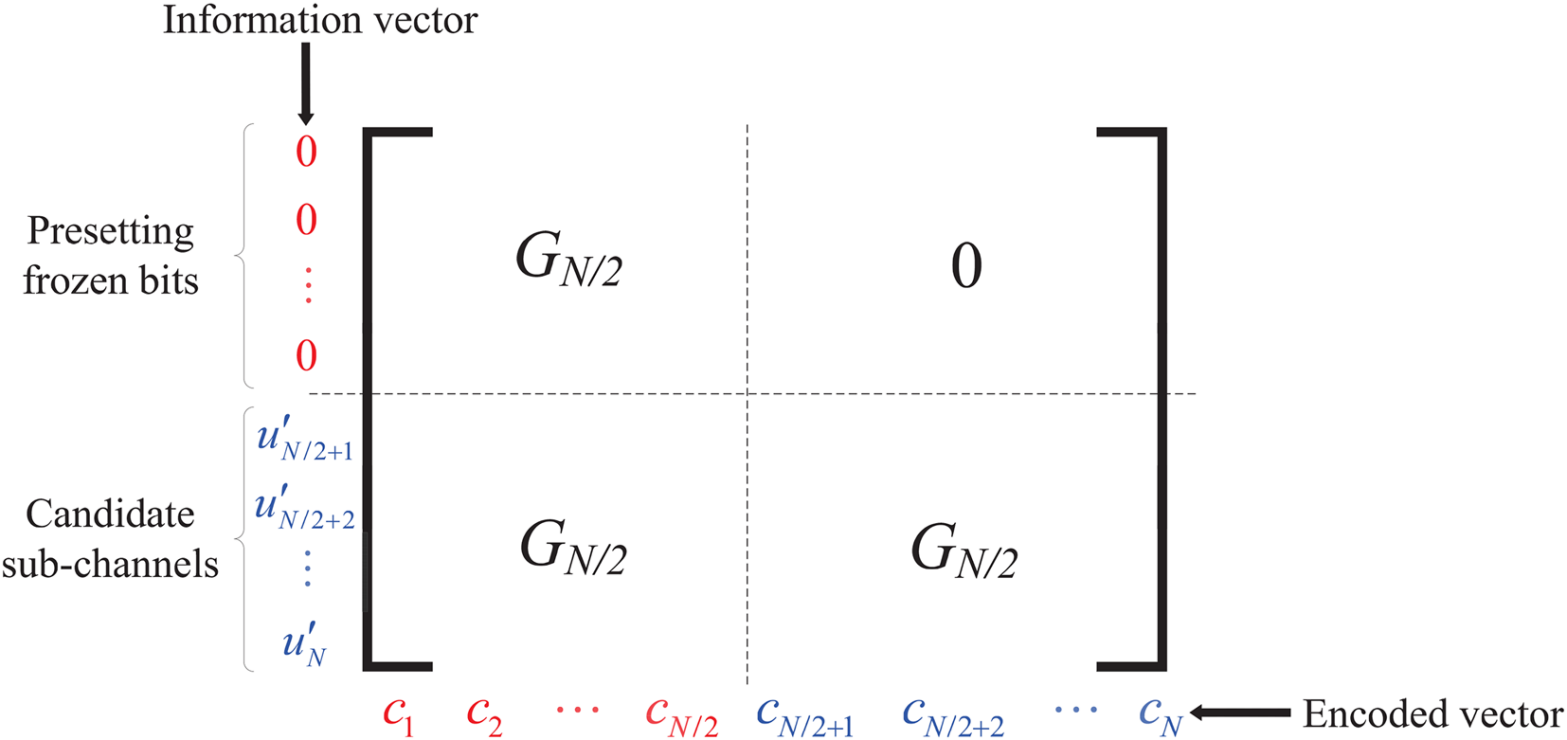
The proposed encoded procedure.

Since the cardinality of ${\mathscr {A}}^{c*}$ is non-negative, the code rate *R* is limited by $0 \le R \le 1/2$. For the presented algorithm, the input vector $u_1^{\prime N}$ is then denoted by $(0,0,\ldots ,0, u_{N/2+1}^{\prime },u_{N/2+2}^{\prime },\ldots ,u_{N}^{\prime } )$. According to the mathematical structure of the generator matrix ${\textbf {G}}_{N}$, we have two symmetric codes, $(c_1, c_2, \ldots , c_{N/2})$ and $(c_{N/2+1}, c_{N/2+2},\ldots , c_N)$, in the encoded vector, where $c_i = c_{i- N/2}$, $N/2 + 1 \le i \le N$. The encoded procedure is shown in Fig. 2.

After transmitting over the channel, the *i*-th and the $(i - N/2)$-th received signals can be added together to form a new combined signal $y_i^{\prime }=(y_i+y_{i-N/2})/2$ for $N/2 + 1 \le i \le N$, which can mitigate the channel interference. Consequently, the ED between $y_i^{\prime }$ and $x_i$ can be calculated as

11 $$\begin{aligned} \begin{aligned} D_i^{\prime }&=||(y_i+y_{i-N/2})/2-x_i||^2\\&=(y_i^{\prime }-(1-2\sum _{j=i}^N\oplus u_j^{\prime }g_{j,i}))^2 , \end{aligned} \end{aligned}$$

where $N/2 + 1 \le i \le N$. Correspondingly, the partial ED value $d_i^{\prime N}$ can be computed as

12 $$\begin{aligned} d_i^{\prime N}=\sum _{k=i}^{N}(y_k^{\prime }-(1-2\sum _{j=k}^N\oplus u_j^{\prime }g_{j,k}))^2, \end{aligned}$$

where $$N/2 + 1 \le i \le N$$.

**Figure 3:**
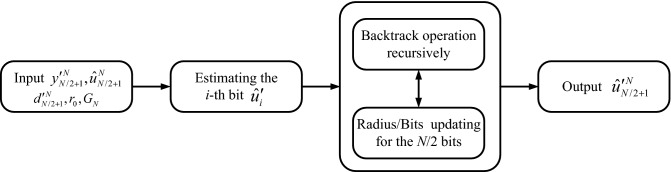
The flow chart of the presented algorithm.

Based on the description above, the presented symmetric-coded based SD algorithm can be summarized in Algorithms 1 and 2. Figure 3 shows the flow chart of the presented algorithm.
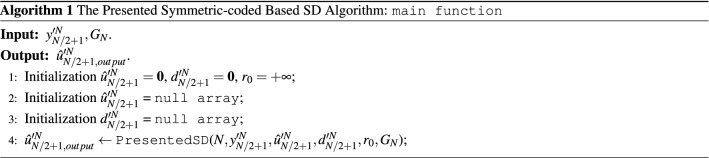


*Remark* Since the former half of the input bits are set to be frozen bits, the SD search only needs to estimate the latter *N*/2 bits, $(u_{N/2+1}^{\prime },u_{N/2+2}^{\prime },\ldots ,u_{N}^{\prime } )$. Correspondingly, the backtrack operation of the algorithm is not required for the former *N*/2 bits, which can significantly reduce the computational complexity. The resulting encoded bits include two symmetric codes, whose received signals can be combined together to compute the ED value, which can mitigate the channel corruption. Such signal combination acts like the repeat code and can compensate the performance degradation caused by the frozen bits presetting.

## Performance and complexity evaluation

In this section, we verify the effectiveness of the proposed algorithm and analyze the computational complexity in terms of the average visited nodes (AVN). As mentioned in the previous section, the resulting code of the presented algorithm is available for moderate-to-low rate with $0 \le R \le 1/2$. Furthermore, polar codes decoded by SD algorithm show advantages in performance for the short block length, which is suitable for the control channel in which the payload size is relatively small. For this reason, polar codes with short block length and low rate are considered in the simulations.

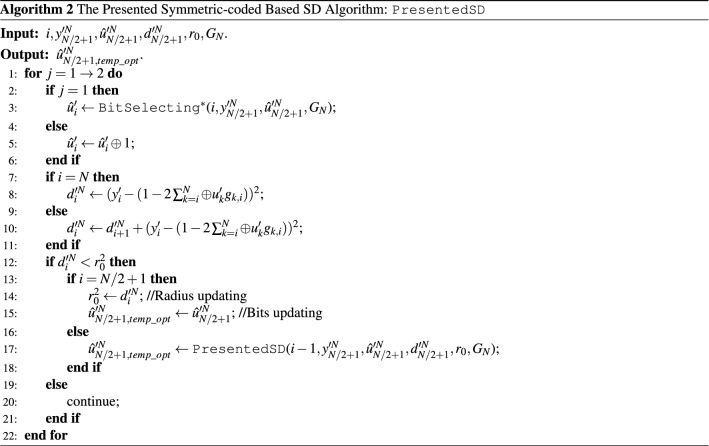



*: BitSelecting:$${\hat{u}_i^{\prime }} = \mathop {argmin}\limits _{u_k^{\prime }\in \{0,1\}}(y_i^{\prime }-(1-2\sum _{k=i}^N\oplus u^{\prime }_kg_{k,i}))^2$$

Consider the polar codes, *P*(64, 8), *P*(64, 10), *P*(64, 12) and *P*(64, 14) with code rate of 4/32, 5/32, 6/32 and 7/32, respectively. For simplicity, we only consider the AWGN channel with BPSK modulation. The total number of simulation frames is $10^6$ and the maximum number of error frames is 2000. When the simulation reaches the total number of frames or reaches the maximum number of error frames, the algorithm stops decoding.

**Figure 4:**
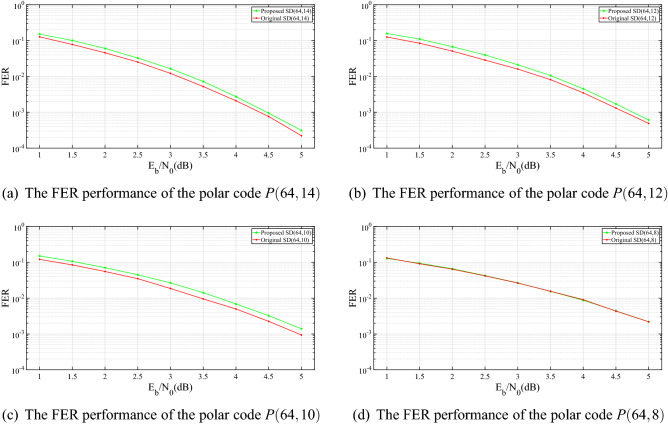
The FER performance of the polar codes with $N=64$ under different rates.

Figure 4 shows the frame error rate (FER) performance of these four codes. We have the following observations.

- The slopes of the performance curves almost remain the same and the error floor is not observed.
- For the polar code *P*(64, 8) with the smallest information bit $K=8$, the presented symmetric-coded based SD algorithm performs as well as the original SD algorithm.
- With the increasing length of information block, the presented symmetric-coded based SD algorithm performs slightly worse compared to the original SD algorithm. For example, the polar codes *P*(64, 10), *P*(64, 12) and *P*(64, 14) with information bits $K=10, 12, 14$ show a performance gap within 0.2 dB compared to the original SD algorithm, which is acceptable.

It is worthwhile to point out that, to construct the symmetric code, the former half of the sub-channels are designated to place the frozen bits. This may cause some problems that have negative influence on performance, since some “good” sub-channels are excluded. Correspondingly, some “bad” sub-channels are included into the information set ${\mathscr {A}}$. Such changed sub-channels proportion $\lambda$ is defined by

$$\begin{aligned} \lambda = \frac{\text {the number of changed sub-channel}}{\text {the half of block length}}. \end{aligned}$$

Fortunately, it is observed that such proportion $\lambda$ only occupies a very small proportion, especially for small *K* value, which has a limited impact on performance. To verify this point, we give the proportion $\lambda$ of the polar codes with block length $N=64$ and different information lengths of $K=8, 12, 14, 16$, respectively, as shown in Table 1. It can be seen that, the proportion $\lambda$ increases with the value of *K*. For example, $\lambda =0$, $3.1 \%$ and $6.2\%$ for $K=8, 12, 16$, respectively.

**Table 1:** The proportion of the changed sub-channels.

Polar codes	*P*(64, 8)	*P*(64, 12)	*P*(64, 14)	*P*(64, 16)
Sub-channels’ number	0	1	1	2
Proportion $\lambda$	0	3.1%	3.1%	6.2%

**Figure 5:**
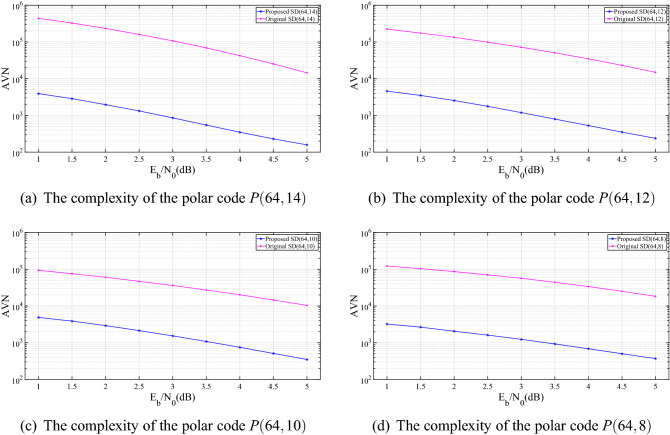
Complexity: the average visited nodes.

Similar to ref. ^11^, the complexity in this paper is analyzed by the average visited nodes during the SD search. Furthermore, the presented SD algorithm is performed with fixed lower bounds reported in ref. ^11^, which can remove some branches at an earlier stage. Figure 5 shows the complexity in terms of AVN for the polar codes *P*(64, 8), *P*(64, 10), *P*(64, 12) and *P*(64, 14). It can be seen from the figure that

- Compared to the original SD algorithm, the presented SD algorithm can significantly reduce the complexity for these four polar codes. The complexity reduction mainly comes from the following two facts: 1)Due to the symmetric code construction, the block length reduces from *N* to *N*/2; 2) Instead of the first bit, the backtrack operation starts from the $(N/2+1)$-th bit, which can dramatically reduce the visited nodes.
- For example, for the polar code *P*(64, 8) and *P*(64, 14) at SNR = 1 dB, the original SD algorithm requires about 120,000 and 440,000 AVN, respectively. In contrast, the presented symmetric-coded based SD algorithm only requires about 3,000 and 4,000 AVN, which is $2.6\%$ and $0.9\%$ to that of the original SD algorithm, respectively.

## Conclusion

In this paper, we have proposed a new variant of the SD algorithm, called the symmetric-coded based SD algorithm, for the short and low-rate polar codes. At the encoding stage, the former half of the input bits are set to be frozen bits and the resulting encoded bits include two symmetric codes. At the decoding stage, the SD search only requires to estimate the latter *N*/2 bits. Meanwhile, received signals with respect to the symmetric code bits can be combined together to compute the ED value, which can mitigate the channel corruption. Furthermore, the presented algorithm starts the backtrack operation from the $(N/2+1)$-th bit instead of the first bit, which can significantly reduce the computational complexity. Simulation results show that, compared to the original sphere decoding, the presented variant of the SD algorithm can reduce the average visited nodes to $0.9\%$ for the polar code *P*(64, 14) at SNR = 1 dB with a performance loss within 0.2 dB.

The presented algorithm only considers codes with short length and low rate, where the changed sub-channels proportion $\lambda$ remains relative small. It is interesting to investigate codes with longer length and higher rate (consequently producing a larger $\lambda$ value which may cause performance degradation) in the future. Furthermore, to jointly design the presented algorithm with the polar-coded MIMO systems may be another valuable research direction.

## Supplementary Information

Supplementary Information.

## Data Availability

The datasets used and/or analysed during the current study available from the corresponding author on reasonable request.
