# Supplementary material for: Low complexity symmetric-coded based sphere decoding for low-rate polar codes
Source: Sci Rep. 2023 Jan 21;13:1191. doi: 10.1038/s41598-023-28256-1 (PMC9867773; doi:10.1038/s41598-023-28256-1)
Supplement: Supplementary file 1 — Supplementary Information. [file 41598_2023_28256_MOESM1_ESM.pdf]

Appendix

Acronyms	Full name	Acronyms	Full name	Acronyms	Full name
SD	Sphere Decoding	ML	Maximum Likelihood	MIMO	Multiple-output Multiple-output
ED	Euclidean distance	eMBB	Enhanced Mobile Broadband	SC	Successive Cancellation
SCL	Successive Cancellation List	CA-SCL	Cyclic Redundancy Check Aided SCL	SISO	Soft-input Soft-output
KSD	King Sphere Decoding	LSD	List Sphere Decoding	LLR	Logarithmic Likelihood Ratio
CA-SD	CRC-Aided SD	DFT	Discrete Fourier Transform	AVN	Average Visited Nodes
GA	Gaussian Approximation	BI-AWGN	Binary Input Additive White Gaussian Noise	FER	Frame Error Rate

Supplementary Table 1. The acronyms
